# Antiviral Effects of Novel 2-Benzoxyl-Phenylpyridine Derivatives

**DOI:** 10.3390/molecules25061409

**Published:** 2020-03-19

**Authors:** Yanhong Wei, Haijie Wang, Caili Xi, Ni Li, Dong Li, Chenguang Yao, Ge Sun, Hongmei Ge, Kanghong Hu, Qian Zhang

**Affiliations:** 1 National “111” Center for Cellular Regulation and Molecular Pharmaceutics, Key Laboratory of Fermentation Engineering (Ministry of Education), Hubei Provincial Cooperative Innovation Center of Industrial Fermentation, Hubei Key Laboratory of Industrial Microbiology, Sino-German Biomedical Center, School of Materials and Chemical Engineering, Hubei University of Technology, Wuhan 430068, China; weiyanhong925@163.com (Y.W.); 13007120460@163.com (H.W.); cailixi0210@163.com (C.X.); 18271835145@163.com (N.L.); mengqingchenguang@163.com (C.Y.); dove1012@126.com (G.S.); esapir@163.com (H.G.); 2 School of Materials and Chemical Engineering, Hubei University of Technology, Wuhan 430068, China; dongli@mail.hbut.edu.cn

**Keywords:** 2-benzoxyl-phenylpyridine derivatives, antiviral activity, CVB3, ADV7

## Abstract

Coxsackievirus B3 (CVB3) is the most common cause of acute and chronic viral myocarditis, primarily in children, while human adenovirus infections represent a significant cause of morbidity and mortality worldwide, in people of all ages. A series of novel 2-benzoxyl-phenylpyridine derivatives were evaluated for their potential antiviral activities against CVB3 and adenovirus type 7 (ADV7). Preliminary assays indicated that some of these compounds exhibited excellent antiviral effects on both CVB3 and ADV7 viruses; they could effectively inhibit virus-induced cytopathic effects, reduce viral progeny yields, and had similar or superior antiviral activities compared with the control drug, ribavirin. Further, these compounds targeted the early stages of CVB3 replication in cells, including viral RNA replication and protein synthesis, rather than inactivating the virus directly, inhibiting virus adsorption/entry, or affecting viral release from cells. Our data demonstrate that the tested 2-benzoxyl-phenylpyridine derivatives are effective inhibitors of CVB3 and ADV7, raising the possibility that these compounds might be feasible candidates for anti-viral agents.

## 1. Introduction

Diseases caused by viral infection are a major public health issue. Coxsackievirus B3 (CVB3) is non-enveloped, single-stranded (+) RNA virus, belonging to the picornavirus family. CVB3 is considered a pathogen of significant importance, as it is the most common cause of acute and chronic viral myocarditis, primarily in children [1]. Moreover, CVB3 is associated with the development of respiratory infections, aseptic meningitis, encephalitis, pancreatitis, hepatitis, and acute episodes of hand, foot, and mouth disease [2,3,4,5]. To date, there is no specific drug approved for the treatment of CVB3 infection [6]. Some reports have presented effective candidates for treatment of CVB3 [7,8], and many molecules that block CVB replication in vitro, attacking different points of the viral life-cycle, have been reported in the past few decades; however, none have received final market approval because of adverse side effects or unsatisfactory antiviral activity [9,10]. Therefore, it is of great interest to search for novel and effective antiviral drug candidates against CVB3.

Adenoviruses (ADV) are double-stranded DNA viruses with an icosahedral protein capsid. Human adenoviruses (HADV) belong to the genus *Mastadenovirus* (Family *Adenoviridae*) and comprise seven species, including over 70 serotypes [11], and human adenovirus infections represent a significant source of morbidity and mortality worldwide, among people of all ages. ADV infection causes fatal acute respiratory distress syndrome in healthy adults and is especially lethal in infants and immune-compromised individuals [11,12,13,14]. ADV7 and ADV3 are the most prevalent forms of ADV in China [15]. Several phosphonyl acyclic nucleotide analogs have been demonstrated to inhibit HADV infection. Cidofovir (a deoxycytosine monophosphate analog) and brincidofovir (CMX001) (a lipid ester form of cidofovir), which target the viral DNA polymerase, have been successfully applied in the clinic; however, they are associated with significant toxicity to the kidney and gastrointestinal tract, respectively [16,17,18]. To date, no safe and effective treatment is available for HADV infections [19], highlighting the urgency and significance of developing suitable anti-ADV agents.

Aromatic esters are widely found in nature and are significant and versatile compounds, which are important ingredients in food, beverages, cosmetics, pharmaceuticals, chemicals, and personal care products, such as perfumes, body lotions, face creams, shampoos, soaps, shower and shaving gels, and other toiletries, due to their flavor and fragrance properties [20]. Their rapid absorption, metabolic excretion, low level of use, and lack of significant genotoxic and methanogenic potential mean that aromatic esters are generally recognized as safe for use as flavor ingredients [21]. The European commission, Food and Drug administration (FDA), and the International Joint Food and Agriculture Organization of the United Nations/World Health Organization Expert Committee on Food Additives have approved these esters as food additives [22,23]. Emerging studies have demonstrated that aromatic esters can have biological activities, including antioxidant, antimicrobial, and larvicidal properties [20,24]. These ester compounds are usually obtained by direct extraction from plant or fruit sources. Chemical synthesis and biotechnological production are alternatives to natural sources and provide the possibility to scale up production for industrial applications. 

In a previous study, we developed a simple synthesis strategy for the construction of novel aromatic ester compounds (2-benzoxyl-phenylpyridine derivatives) [25]. Here, we investigated these compounds for their antiviral properties against CVB3 and ADV7 infection in vitro. The ultimate aim of this study was to uncover new information about the scope of the biological activity of these novel 2-benzoxyl-phenylpyridine derivatives.

## 2. Results

### 2.1. Antiviral Activities of Novel 2-Benzoxyl-Phenylpyridine Derivatives against CVB3 and ADV7

**Figure 1:**
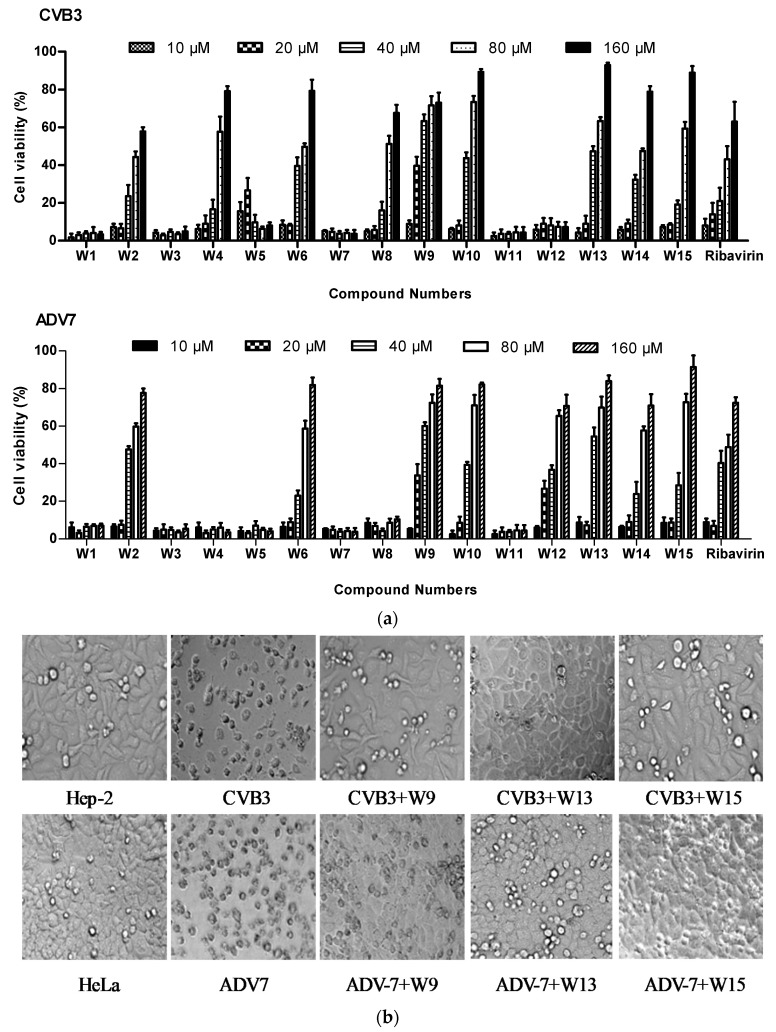
Antiviral activities of novel 2-benzoxyl-phenylpyridine derivatives against CVB3 and ADV7. (**a**) The compounds inhibited virus-induced cytopathic effects (CPEs). Hep-2 or HeLa cells were infected with 100 TCID_50_ of CVB3 or ADV7 for 1.5 h at 37 °C. Then, the inocula were aspirated and the cells were incubated with various concentrations of test compounds for 48 h. Cell viability was determined using the MTT assay. (**b**) Images showing the morphology of Hep-2 and HeLa cells treated with 120 μM W-9, 160 μM W-13, or 160 μM W-15 (magnification, 20×).

A series of 2-benzoxyl-phenylpyridine derivatives were tested against CVB3 and ADV7 in cell-based assays. Cytotoxicity and antiviral activity were evaluated in parallel, using ribavirin as a reference inhibitor. The inhibitory activities of all tested compounds, expressed as half-maximal effective concentration (EC50) values and selectivity indices, are presented in Table 1. Dose-dependent antiviral effects are shown in Figure 1a. Most tested compounds exhibited low cytotoxicity in Hep-2 and HeLa cells, with average 50% cytotoxic concentration (CC50) values of 324.8 and 637.5 μM, respectively (Table 1). Further, the tested compounds displayed moderate to good inhibitory activities against CVB3 and ADV7. The EC50 values for the active compounds against CVB3 ranged from 24.6 to 100.7 μM, with values of 27.1 to 75.4 μM against ADV7, which were comparable or better than those of the control compound, ribavirin (Table 1). Notably, compounds W2, W6, W9, W10, W13, W14, and W15 displayed significant inhibitory activities against CVB3 and ADV7 infections. W4 and W8 exhibited selective antiviral activities against CVB3, while W12 showed selective anti-ADV7 activity. In particular, W9, W13, and W15 showed the most potent inhibitory activities, with 73%, 92.8%, and 90% inhibition of CVB3 infections, and 81.4%, 83.9%, and 89.4% inhibition of ADV7 infections, respectively, at 160 μM (Figure 1a). In comparison, the maximum inhibition values of CVB3 and ADV7 by ribavirin were 63.0% and 72.4%, respectively, indicating that the compounds W9, W13, and W15 exhibited stronger inhibitory effects than ribavirin. Overall, these results show that some of the tested compounds exhibit potent antiviral activities against CVB3 and ADV7, with low toxicity to host cells.

Further, we found that different substituents at the periphery of these molecules could lead to clear differences in activity (Table 1). In most of tested compounds (W-2, W-9, W-10, W-14, and W-15), substitution of position 4 in the central benzene ring led to more potent antiviral activities than substitutions at other positions, and the antiviral activities of the compounds could be attenuated by inclusion of a substituent on the periphery of the nitrogen-containing heterocycle (W-4, W-8, and W-12). Among these compounds, W9 and W10 were clearly endowed with superior antiviral activities against both viruses relative to W4 and W12 with the same substituents (Cl and Br). Furthermore, compounds containing substituted groups at position 5 of the central benzene ring also showed excellent antiviral activities (W-6 and W-13, but not W-5). These data suggest that the presence substituted groups at positions 4- or 5- in the central benzene ring may be crucial for antiviral activity, which may inform the design of more powerful antiviral compounds in the future.

Since CVB3 and ADV7 infections cause marked cytopathic effects (CPEs), we evaluated the antiviral properties of these compounds by analysis of CPEs in Hep-2 and HeLa cells infected with CVB3 and ADV7, respectively. Inhibition of virus-induced CPEs by the representative compounds, W9, W13, and W15, are shown in Figure 1b. Virus-infected cells appeared rounded and detached from the dish in the absence of test compounds, whereas treatment with 120 μM W-9, 160 μM W-13, or 160 μM W-15, produced almost complete protection against virus-induced CPEs. These results show that virus infection causes obvious CPEs in host cells, which can be significantly ameliorated by the tested compounds, indirectly demonstrating the potent inhibitory activities of the compounds against virus infection.

### 2.2. Effects of Test Compounds on Virus Progeny Yields

**Figure 2:**
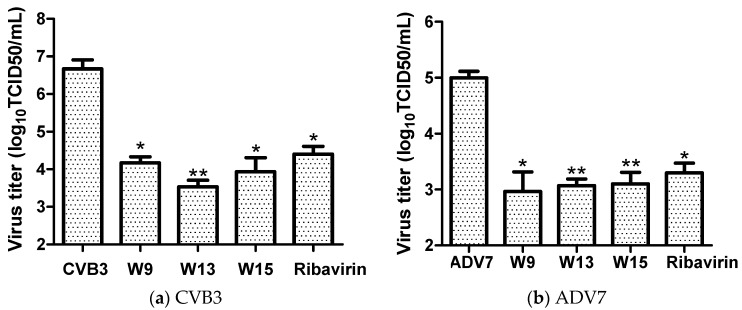
The effects of W9, W13, and W15 on progeny virus production. Hep-2 cells infected with 100 TCID_50_ of CVB3 (**a**), or HeLa cells infected with 100 TCID_50_ of ADV7(**b**), were incubated in the absence (virus control) or presence of 120 μM W-9, 160 μM W-13, 160 μM W-15, or 160 μM ribavirin for 36 h, and the culture media and cell lysates then harvested for virus titration. Viral titers are presented as Log_10_ TCID_50_/mL. * *p* < 0.05; ** *p* < 0.01, compared with the virus control group.

Based on their antiviral activities and therapeutic selectivity indices, the effects on virus progeny yields of compounds W9, W13, and W15 were further investigated, using ribavirin as a positive control drug. To this end, confluent monolayers of Hep-2 or HeLa cells in 96-well plates were infected with 100 × median tissue culture infective dose (TCID_50_) of CVB3 or ADV7, then treated with 120 μM W-9, 160 μM W-13, 160 μM W-15, or 160 μM ribavirin. After 36 h, culture media and cell lysates were collected, following freeze-thaw cycles, and then subjected to virus titration. As shown in Figure 2, treatment with W9, W13, or W15 resulted in significant reductions in CVB3 and ADV7 virus progeny titers of approximately 2.5 and 2.0 logs, respectively, relative to untreated controls. The inhibitory activities of the tested compounds on virus progeny production were also superior to those of the positive control drug, ribavirin, which resulted in an approximately 2.0 log reduction for CVB3 and 1.7 log reduction for ADV7. These data demonstrate that the compounds, W-9, W-13, and W-15, are potent inhibitors of viral proliferation in host cells.

### 2.3. Preliminary Studies of the Mechanism(s) of Action of the Compounds against CVB3

To determine whether these 2-benzoxyl-phenylpyridine derivatives inactivated virions directly, 10^4^ TCID_50_ of CVB3 suspension was incubated with 120 μM W-9, 160 μM W-13, 160 μM W-15, or 160 μM ribavirin for 2 h at 37 °C. Subsequently, viral titers were measured by inoculating 100-fold dilutions of the mixtures, beyond the effective concentrations of the compounds, into the host cells. TCID_50_ values were calculated by the Reed–Muench method on day 2 post-inoculation. No significant difference was found between virus titers of the mixture for CVB3 with or without the test compounds (data not shown), suggesting that none of the compounds showed viricidal activity against CVB3.

**Figure 3:**
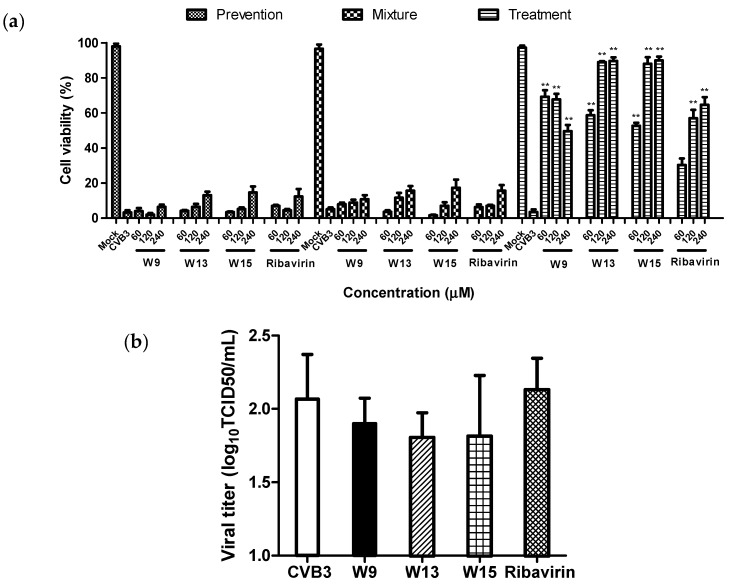

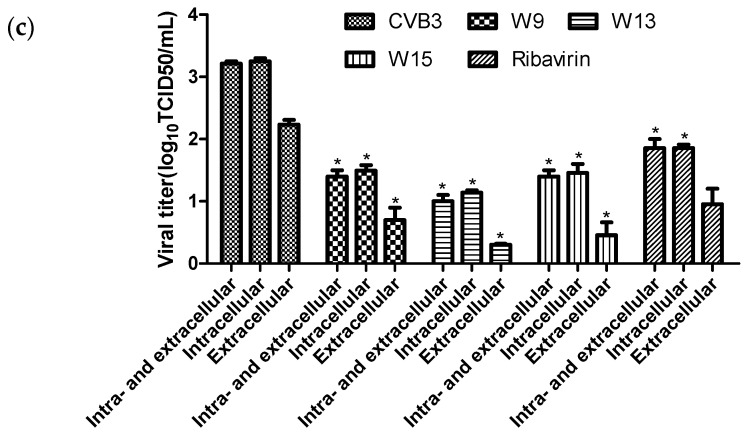
Analysis of the modes of action of W9, W13, and W15 against CVB3. (**a**) Analysis of effective stage. The antiviral effects of test compounds against CVB3 were detected by measuring cell viability after 48 h of infection when cells were treated with W9, W13, or W15 before, simultaneously, or after inoculation with CVB3 (100 TCID_50_). (**b**) Analysis of the effects on CVB3 adsorption. Mock-, 120 μM W-9-, 160 μM W-13-, 160 μM W-15-, or 160 μM ribavirin-treated CVB3 (10^6^ TCID_50_) was inoculated onto Hep-2 cells and adsorbed for 2 h. Infected cells were then harvested and subjected to virus titration, using the TCID_50_ method. (**c**) The effects of test compounds on CVB3 release from Hep-2 cells. Hep-2 cells infected with 100 TCID_50_ of CVB3 were incubated with test compounds for 12 h, then cells and culture supernatants (intra- and extracellular) analyzed for virus yield, separately or together. Mock, no infection; VC, virus control. Values represent the means ± SDs of three independent experiments. * *p* < 0.05; ** *p* < 0.01, compared with the virus control group.

The mechanisms by which W9, W13, and W15 inhibit CVB3 infection were further investigated by three different approaches, using ribavirin as a positive control. Antiviral effects against CVB3 were detected by measuring cell viability after 48 h of infection when cells were treated with compounds W9, W13, and W15 before, simultaneously, or after inoculation with CVB3 (100 TCID_50_). As shown in Figure 3a, all of the tested compounds exhibited the most powerful inhibitory effects against CVB3 post infection, while no significant inhibition was detected when the compounds were added just before or during infection. These results indicate that W9, W13, and W15 do not exhibit preventive effects against CVB3, nor do they interact with the viral particles to prevent adsorption/entry of CVB3, rather, they mainly suppress viral replication within host cells.

The conclusion that the compounds do not act to inhibit adsorption/entry was further confirmed by direct analysis of adsorption/entry. To this end, Hep-2 cells were mock-treated or inoculated with CVB3 (10^6^ TCID_50_) treated with 120 μM W-9, 160 μM W-13, 160 μM W-15, or 160 μM ribavirin, and adsorbed for 2 h. Infected cells were then harvested and subjected to virus titration, using the TCID_50_ method. As shown in Figure 3b, no significant decrease in virus titer was detected during virus attachment in the presence of W9, W13, W15, or ribavirin, confirming that virus adsorption/entry is not quantitatively affected by any of these compounds. These findings are consistent with the conclusions obtained by analysis of effective stage, and further confirm that these compounds do not influence the adsorption/entry stage of the viruses.

The viral life cycle is completed by release of progeny for further infection of neighboring cells. Therefore, we tested whether viral release from cells is affected by W9, W13, and W15. Hep-2 cells infected with 100 TCID_50_ of CVB3 were incubated with test compounds for 12 h, then both cells and supernatants (intra- and extracellular), or one of which was harvested separately, and virus yields were determined. Ribavirin was used as the positive control. Figure 3c shows that the virus titers from infected Hep-2 cells, culture supernatants, or total solutions (cell lysates and supernatants) treated with test compounds were significantly reduced, with approximately the same inhibition levels observed in each, implying that CVB3 release was unaffected. These results indicate that these compounds, including the positive control, ribavirin, show significant antiviral activity against CVB3, but do not act on viral release from cells.

### 2.4. W9, W13, and W15 Affect the Early Stages of Viral Replication in Cells

**Figure 4:**
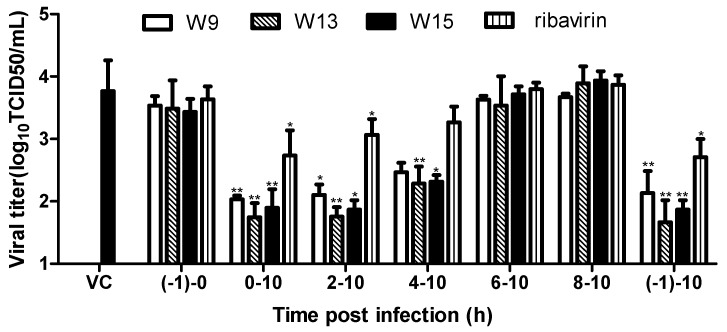
Time-of-addition assay. 120 μM W-9, or 160 μM W-13, W-15, or ribavirin, were added to Hep-2 cells for different time periods after CVB3 infection. Progeny virus yield was determined at 12 h post-infection ((−1)–0 h, viral infection period; 0–10 h, period of virus proliferation in cells). Values are presented as means ± SDs. * *p* < 0.05; ** *p* < 0.01, compared with the virus control group.

To further elucidate the mechanisms underlying the activities of W9, W13, and W15 against CVB3 propagation in cells, we conducted a time-of-addition experiment. Briefly, 120 μM W-9, or 160 μM W-13, W-15 or ribavirin, were added to Hep-2 cells at different time periods following CVB3 infection and progeny virus yield was determined at 12 h post-infection (pi). As shown in Figure 4, when the tested compounds were present for the entire course of the replication cycle (−1–10), progeny virus titers were reduced, to similar levels observed on addition of drugs during the 0–10 and 2–10 h stages after viral infection. In contrast, when compounds were added during the 4–10 h stage after viral infection, production of viral progeny increased somewhat, however, compared with the virus control group, the inhibition effect was still clear. Drug treatments during other periods following CVB3 infection led to a gradual increase in viral yields, reflecting a loss of antiviral effects. The control compound, ribavirin, had very similar effects. These data indicate that the test compounds may exert their anti-CVB3 effects through interference with early viral replication events.

### 2.5. W13 Strongly Inhibits CVB3 Replication in Hep-2 Cells

**Figure 5:**
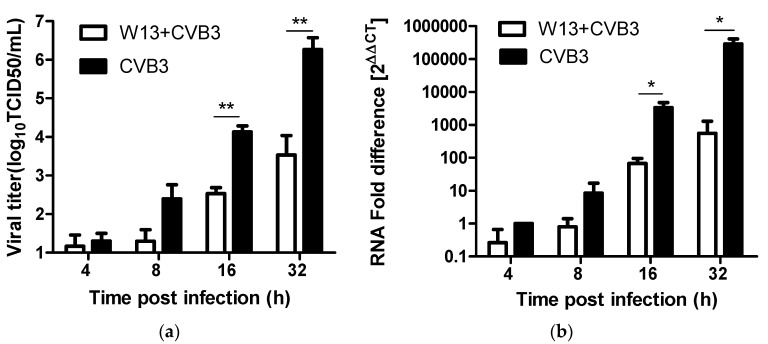

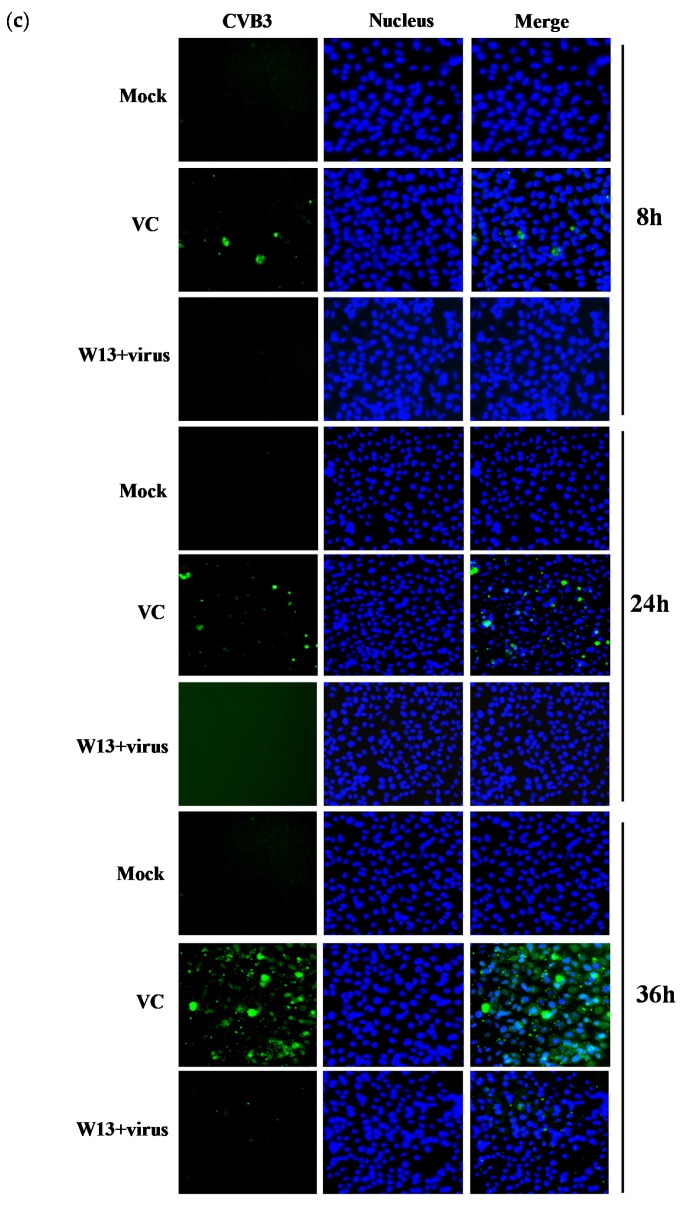
Effect of W13 on CVB3 replication in Hep-2 cells. Hep-2 cells infected with 100 TCID_50_ CVB3 were incubated in the absence or presence of 160 µM W13 and harvested at the indicated times post-infection. (**a**) Progeny virus yields were determined. (**b**) Viral RNA levels were measured by analysis of total RNA. GAPDH was amplified and the resulting data was used for normalization. Values represent the means ± SDs of three independent experiments. * *p* < 0.05; ** *p* < 0.01, compared with the virus control group. (**c**) CVB3 protein levels were determined by indirect immunofluorescence, using the mouse anti-coxsackie virus B3 monoclonal antibody, MAB948, and an alexa-fluor-488-conjugated affiniPure goat anti-mouse IgG (H + L), after incubation with W13 for 24 h. Nuclei were stained with DAPI and green foci indicate the presence of CVB3 protein.

To further investigate the effects of these compounds on CVB3 replication, we analyzed the efficacy of representative compound, W13, in inhibiting viral progeny yield, RNA synthesis, and protein translation. Infected cells treated with or without 160 μM W-13 were harvested after 4 h, 8 h, 16 h and 32 h, viral progeny yields were determined using the Reed–Muench method, and quantitative reverse transcription polymerase chain reaction analysis of harvested cells were conducted, to evaluate the relative amounts of viral RNA. As shown in Figure 5a, virus titers continued to increase from 4 to 32 h in control virus-infected cells, indicating active viral replication in the cells following viral inoculation. Notably, less change was observed in cells treated with W-13, and the inhibitory effect was most prominent at 32 h pi, with an approximate 3.0-log reduction in viral replication. Figure 5b illustrates the efficacy of W-13 in inhibiting viral RNA synthesis. Viral RNA became detectable in the virus control group during the first 4 h pi, followed by significant increases at subsequent time points. Viral copy numbers in W-13-treated Hep-2 cells were significantly lower than those in virus-infected control cells, and the most prominent inhibition (approximately 500-fold reduction) was also observed at 32 h pi. These results indicate that W-13 can strongly inhibit viral replication in the cells and, over time, inhibition gradually accumulates, leading to increasingly strong inhibitory effects.

The influence of W13 on CVB3 replication was also determined at the translational level. Hep-2 cells grown on 24 coverslips were infected with CVB3 and treated with 160 μM W13. Then, intracellular viral protein was detected by indirect immunofluorescence analysis after incubation for 8, 24, and 36 h. Figure 5c shows that immunofluorescence foci of viral proteins were not observed in mock-infected control cells, suggesting that the antibody was specific for CVB3. Further, green immunofluorescence foci in the virus control group were significantly more abundant than those in W13-treated cells, indicating that viral protein synthesis was suppressed by W13. These results lead us to propose that W13 suppresses CVB3 replication by inhibiting viral RNA and protein synthesis.

## 3. Discussion

CVB3 has been consistently reported as the predominant pathogen causing human viral myocarditis, and current treatment approaches for patients with CVB3-related myocarditis are almost entirely supportive, as there is no vaccine or specific treatment for infections caused by coxsackieviruses [26]. In addition, HADV are the most common infectious cause of ocular disease worldwide, and HADV infections are associated with considerable morbidity and mortality, which could clearly be limited by the availability of effective antiviral drugs. Moreover, the increased clinical use of HADV-derived replication-competent vectors makes the availability of effective antivirals against HADV infections desirable [27]. To date, no FDA-approved antiviral for treatment of HADV infections is available [28]. We provide evidence that a series of 2-benzoxyl-phenylpyridine derivatives show strong antiviral activity, and may represent novel antiviral agents. Aromatic esters are broadly present in nature and used for a great variety of applications in the food, pharmaceuticals, and cosmetic industries. These compounds also have emollient, surfactant, and antioxidant properties [20]. This study is the first to test the antiviral activities of these novel aromatic ester compounds. There are few reports on the inhibition of viral infection by compounds with similar structures. For example, derivatives of the metal ion chelator, quercetin, are reported to show potent anti-HCV activities [29]. Therefore, our research provides new information applicable to the development of active aromatic ester compounds.

Our assay of antiviral effects against CVB3 and ADV7 was conducted by microscopic observation of CPEs and determination of cell viability using MTT assays, as well as virus yield reduction assays. CPEs can be reflected in the surface morphology of infected cells, while the MTT assay measures the level of cellular metabolism, and virus yield assays test viral multiplication directly in infected cells. These various analytical methods demonstrated that the test compounds have strong antiviral activity. Moreover, the compounds showed potent post-exposure activity on CVB3-infected cells, as initially tested by effective stage analysis, and confirmed by assessment of viral RNA and protein synthesis inhibition. These experiments comprehensively demonstrate that the test compounds have strong and effective antiviral activity and may be suitable for use as therapeutic agents against viral infection.

High activity, combined with low toxicity, of antiviral candidates are major indicators for the evaluation of application potential. Compounds with selectivity index values ≥ 4 are considered suitable as antiviral agents [30]. W-3, W-13, and W-15 had selectivity indices of 6.0, 10.2, and 6.5 against CVB3, and 9.8, > 10, and 12.2 against ADV7 infections, respectively, which were comparable or superior therapeutic effects to those of ribavirin (8.3 for CVB3 and 7.1 for ADV7), suggesting that these compounds may be effective antiviral agents, with relatively safe profiles. In addition, due to their low toxicities and unique flavor characteristics, aromatic ester compounds have been used as ingredients in various fields, including food, medicine, and cosmetics, etc., giving them a unique advantage over other antiviral candidates. These results suggest that the test compounds have potential for use in therapeutic applications against CVB3 and ADV7 infection.

The CVB3 replication cycle can be divided into the following steps: viral attachment, entry, polyprotein translation and cleavage, viral RNA replication, assembly, and release [2]. These critical steps are considered targets for development of antiviral agents [31]. Picornavirus can complete its life cycle in 5–10 h (approximately 8 h). Upon virus absorption and entry into the host cell, the viral genome is translated into viral protein, initially synthesized as a large polypeptide that is subsequently cleaved into individual structural and nonstructural proteins; negative RNA intermediates of the viral genome are also generated to serve as templates for synthesis of multiple positive-strand progeny genomes. These events have been predicted to reach high levels at 3–4 h pi. Progeny virions are then self-assembled from the synthesized viral proteins and RNA genomes. This process begins in the cytoplasm at 4–6 h, and the release of virus particles occurs at 6–10 h [32]. Our time-addition assays of W-9, W-13, and W-15 demonstrated that inhibition of virus yields declined when Hep-2 cells were treated with these compounds later than 4 h pi. Simultaneously, these compounds showed strong activity against viral RNA and protein synthesis, consistent with the high levels of CVB3 genome replication and protein synthesis at 3–4 h post-viral infection. Based on these results, we conclude that the test compounds mainly target the early replication stage of viral RNA replication and protein synthesis, but do not influence viral absorption/entry, assembly or release. Moreover, these compounds have strong, but inconsistent, antiviral activities against CVB3 and ADV7, for example, compounds W4, W8, and W12 had different antiviral activities against the two viruses. Notably, Coxsackie B virus shares a receptor with human adenovirus, referred to as coxsackie/adenovirus receptor, when infecting host cells [33], suggesting that these compounds are unlikely to play an antiviral role by blocking virus absorption/entry, and consistent with our conclusion that they strongly inhibit viral replication within cells. 

Small-molecular-weight compounds with antiviral activity can act by inhibiting viral or host cell molecules that are required for virus replication. Although drugs directed at viral molecules are more virus-specific, they can easily lead to the selection of resistant mutants. By targeting host cell molecules, resistance is less likely to occur, and if the target molecules are necessary for replication of a variety of different viruses, broad-spectrum antiviral activity can be achieved [34]. In this study, we examined the inhibition of CVB3 and ADV7 by novel 2-benzoxyl-phenylpyridine derivatives. CVB3 is a non-enveloped positive-stranded RNA virus, upon infection, its RNA is translated and then transcribed [2]. ADV7 is a non-enveloped icosahedral virus that replicates in the nucleus. Adenoviruses rely heavily on host RNA splicing machinery to express the full complement of viral proteins [35]. Here, we demonstrate that many novel 2-benzoxyl-phenylpyridine derivatives display potent antiviral activities against both CVB3 and ADV7 at noncytotoxic concentrations. These results suggest that the inhibitory mechanisms of the compounds against viral infection are more likely to target cellular molecules than the virus itself. Detailed tests are needed to fully understand the antiviral modes of action of these compounds.

Ribavirin, a broad-spectrum antiviral, is sometimes used to treat patients infected with CVB3 [36,37]. W-9, W-13, and W-15 had greater antiviral activity than ribavirin, while similar results were obtained for all of these compounds in experiments to determine their antiviral mechanisms of action. Although ribavirin was discovered in 1972, its mechanism of action has remained unclear until recently [38]. Several possible mechanisms of action have been proposed for ribavirin, including: (a) inhibition of inosine monophosphate dehydrogenase [39]; (b) inhibition of proinflammatory mediators induced by viral infection [40]; and (c) inducement of lethal mutagenesis after incorporation during viral RNA synthesis, which leads to loss of whole viral RNA genomes [41]. All these theories indicate that ribavirin plays a role in the process of virus replication in cells, as do the compounds investigated in this study; however, the viral inhibition mechanism(s) used by these compounds require further investigation.

Taken together, we demonstrate that a series of 2-benzoxyl-phenylpyridine derivatives are potent inhibitors of CVB3 and ADV7 and mainly target viruses post-infection. These findings provide novel information with potential for application in the development of antiviral drugs, and highlight original functions of novel aromatic ester compounds as antiviral agents.

## 4. Materials and Methods

### 4.1. Cells, Viruses, and Tested Compounds

Human laryngeal carcinoma cells (Hep-2) and human cervical carcinoma cells (HeLa) were maintained in DMEM (Gibco) supplemented with 10% fetal bovine serum (FBS; Gibco), 100 U/mL penicillin and streptomycin, and 2 mM L-glutamine.

CVB3 and ADV7 were propagated in Hep-2 and HeLa cells. Viral titers were determined using the standard method of median tissue culture infective dose (TCID_50_) on corresponding host cells [42]. Ribavirin, used as a positive control, was purchased from Sigma Chemical Co. 2-benzoxyl-phenylpyridine derivatives (W1-W15) were synthesized in our laboratory [25]. Stock solutions of drugs were prepared in dimethyl sulfoxide at a final concentration of 0.1% and diluted with maintenance medium (MM), consisting of DMEM supplemented with 2% FBS.

### 4.2. Antiviral Assays and Selectivity Index

The antiviral activities of compounds against CVB3 and ADV7 were determined by measuring their inhibition of virus-induced CPEs in acutely infected Hep-2 and HeLa cells, respectively. Confluent cell monolayers in 96-well plates were infected with 100 TCID_50_ of CVB3 or ADV7 for 1.5 h at 37 °C, inocula were aspirated, and the cells then were incubated with various concentrations of compounds at 37 °C, 5% CO_2_, for 48 h. CPEs were observed by microscopy and the viability of cells was determined using MTT assays [43]. The concentrations of test compounds required to achieve 50% protection from virus-induced cytopathogenicity (EC50 values) were determined. Adverse effects of tested compounds on host cells were also assessed using the MTT method after exposing uninfected cells to various concentrations of test compounds for 48 h at 37 °C. The CC50 values of compounds were calculated using SPSS software and selectivity indices were the ratio of CC50:EC50. Each experiment was performed in triplicate and at least three times, independently.

### 4.3. Progeny Virus Titration

CVB3 or ADV7 suspensions, serially diluted 10-fold with DMEM containing 2% FBS (DMEM maintenance medium, MM), were inoculated to Hep-2 or HeLa cells in 96-well plates. After 1.5 h incubation at 37 °C in 5% CO_2_, unbound virus was washed out and the MM was added to the cells. After 2 days, infected cells were monitored for CPEs and virus titers were calculated using the Reed–Muench method [42].

### 4.4. Analysis of Effective Stage

To identify the step in the life cycle of CVB3 that was affected by the compounds, various concentrations of W9, W13, and W15 were added to Hep-2 cells, and the following three treatment procedures conducted: (i) To evaluate preventive effects (prevention mode of action), various concentrations of compounds were added to cells for 2 h at 37 °C, followed by washing with MM before virus infection; (ii) To analyze for inhibition of adsorption (mixture mode of action), a mixture of compound and virus was added to cells for 2 h at 37 °C, followed by washing with MM; (iii) To assess therapeutic effects (treatment mode of action), cells were first infected for 2 h at 37 °C, followed by washing with MM, addition of the compounds, and incubation for the duration of the experiment. For all procedures, cell viability was estimated after 48 h of infection, using MTT assays.

### 4.5. Viral Adsorption/entry Analysis

Cells were infected with CVB3 (10^6^ TCID_50_) containing 120 μM W-9, 160 μM W-13, 160 μM W-15, or 160 μM ribavirin, after 2 h adsorption at 37 °C, inocula were discarded, and the cells were washed three times with phosphate buffered saline (PBS), harvested, following three freeze-thaw cycles, and subjected to virus titration. Cells infected with CVB3 in the absence of test compounds were used as untreated virus-infected controls.

### 4.6. Viral Release Analysis

Hep-2 cells were treated with or without 120 μM W-9, 160 μM W-13, 160 μM W-15, or 160 μM ribavirin after infection with 100 TCID_50_ of CVB3. Supernatants and cells were harvested together or separately for the determination of progeny virus yields at 12 h pi by the Reed–Muench method [43].

### 4.7. Time of (drug) Addition Experiment

Hep-2 cells were infected with 100 TCID_50_ of CVB3 and then 120 μM W-9, 160 μM W-13, 160 μM W-15, or 160 μM ribavirin were added for different time periods (−1–0, 0–10, 2–10, 4–10, 6–10, 8–10, or −1–10 h pi, where –1–0 h is the viral infection period and 0–10 h is the period of virus proliferation in the cells). Cells and culture supernatants were harvested at 12 h pi and subjected to three freeze-thaw cycles, after which virus titers were determined by the Reed–Muench method [42].

### 4.8. RNA Extraction and Quantitative Reverse Transcription-PCR

CVB3 RNA was extracted from infected cells and culture supernatants using Trizol (Invitrogen, Carlsbad, CA, USA) and reverse-transcribed using a PrimeScript RT reagent kit (Takara, Tokyo, Japan) according to the manufacturer’s instructions. The products of reverse transcription were quantified using the SYBR Premix Ex Taq II (perfect real time) kit (Takara) and detected with a Step One Plus sequence detection system (Applied Biosystems, Foster City, CA, USA). Expression of GAPDH was used as an internal standard. The specific primer sequences were: CVB3-F, 5′-CGG TAC CTT TGT GCG CCT GTT-3′ and CVB3-R, 5′-GCG GTG CTC ATC GAC CTGA-3′; and GAPDH-F, 5′-GCA CCG TCA CGG CTG AGA AC-3′ and GAPDH-R, 5′-TGG TGA AGA CGC CAG TGG A-3′.

### 4.9. Immunofluorescence Microscopy

Hep-2 cells (1 × 10^5^) were seeded in 24-well plates and incubated overnight. Cells were infected with 100 TCID_50_ of CVB3 for 1.5 h and then treated with or without 160 μM W-13. Wells were fixed with 4% paraformaldehyde for 30 min at room temperature at 24 h pi, then incubated with blocking solution (0.5% bovine serum albumin in PBS) for 1 h at room temperature and reacted with mouse anti-Coxsackie virus B3 monoclonal antibody MAB948 (Millipore, Bedford, MA, USA), diluted in blocking solution, overnight at 4 °C. After the plates had been washed three times with 0.1% Tween-20 in PBS, they were incubated with the appropriate Alexa-Fluor-488-labeled secondary antibody (Invitrogen) and fluorescence evaluated using an OLYMPUS IX73 microscope (Tokyo, Japan).

### 4.10. Statistical Analysis

Experimental results are expressed as means of at least three independent experiments. Values are expressed as means ± standard deviations (SD). Comparisons between experimental and control groups were performed using the unpaired Student’s t-test. In all cases, *p* < 0.05 was considered significant.

## 5. Conclusions

In this study, we demonstrate that many novel 2-benzoxyl-phenylpyridine derivatives display clear and strong antiviral activities against CVB3 and ADV7, with low toxicity to host cells. Preliminary analysis of their mechanism(s) of action indicates that these compounds exert potent post-exposure activity on CVB3-infected cells and mainly target the early stage of viral replication. These data demonstrate that the tested 2-benzoxyl-phenylpyridine derivatives have the potential to be developed as novel therapeutic agents for treatment of viral infections.

## Figures and Tables

**Table 1:** Cytotoxicity and antiviral activity of novel 2-benzoxyl-phenylpyridine derivatives against Coxsackievirus B3 (CVB3) and adenovirus type 7 (ADV7).

Compound Structure	Compound Number	CVB3			ADV7		
		EC50 ^a^ (μM)	CC50 ^b^ (μM)	SI ^c^	EC50 (μM)	CC50 (μM)	SI
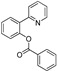	W1	-	594.2 ± 49.1^d^	-	-	1057.1 ± 93.5	-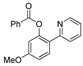	W2	100.7 ± 14.1	240.3 ± 22.0	2.4	50.8 ± 16.7	>655.7	>12.9
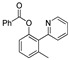	W3	-	630.4 ± 39.4	-	-	920.8 ± 59.5	-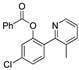	W4	54.4 ± 11.1	221.6 ± 24.1	4.1	-	796.9 ± 45.7	-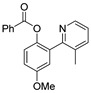	W5	-	33.5 ± 9.4	-	-	38.9 ± 18.8	-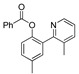	W6	88.4 ± 19.5	243.9 ± 30.4	2.8	66.0 ± 20.1	325.7 ± 21.8	4.9
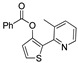	W7	-	349.5 ± 28.8	-	-	1029.8 ± 63.1	-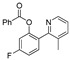	W8	80.1 ± 15.0	354.4 ± 49.5	4.4	-	842.7 ± 37.1	-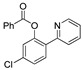	W9	24.6 ± 8.7	148.0 ± 11.6	6.0	27.1 ± 9.0	265.3 ± 28.1	9.8
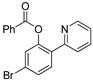	W10	36.7 ± 15.8	184.2 ± 26.3	5.0	51.1 ± 20.1	249.2 ± 23.2	4.9
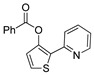	W11	-	378.3 ± 19.2	-	-	1023.8 ± 72.6	-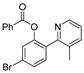	W12	-	128.0 ± 17.9	-	63.6 ± 16.6	211.4 ± 14.7	3.3
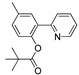	W13	41.6 ± 11.5	423.8 ± 26.8	10.2	36.4 ± 16.4	>371.7	>10
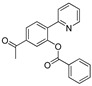	W14	87.1 ± 21.1	490.5 ± 26.8	5.6	75.4 ± 20.2	979.2 ± 27.8	13.0
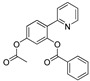	W15	69.7 ± 15.0	451.4 ± 32.4	6.5	65.2 ± 21.6	794.9 ± 33.8	12.2
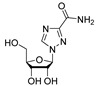	Ribavirin ^e^	101.2	836.5	8.3	114.3	806.6	7.1

^a^ EC50: compound concentration required to achieve 50% protection from virus-induced cytopathogenicity; ^b^ CC50: compound concentration required to reduce cell viability by 50%; ^c^ SI (selectivity index): ratio by CC50/EC50; ^d^ Values represent the mean ± SD of three independent experiments; ^e^ Ribavirin, used as a positive control.

## References

[1] Massilamany C., Gangaplara A., Reddy J. (2014). Intricacies of cardiac damage in coxsackievirus B3 infection: Implications for therapy. Int. J. Cardiol..

[2] Garmaroudi F.S., Marchant D., Hendry R., Luo H., Yang D., Ye X., Shi J., McManus B.M. (2015). Coxsackievirus B3 replication and pathogenesis. Future Microbiol..

[3] Huber S., Ramsingh A.I. (2004). Coxsackievirus-induced pancreatitis. Viral Immunol..

[4] Kemball C.C., Flynn C.T., Hosking M.P., Botten J., WhittonJ L. (2012). Wild-type coxsackievirus infection dramatically alters the abundance, heterogeneity, and immunostimulatory capacity of conventional dendritic cells in vivo. Virology.

[5] Ni H.X., Yi B., Yin J.H., Fang T., He T.F., Du Y., Wang J., Zhang H.W., Xie L., Ding Y.B. (2012). Epidemiological and etiological characteristics of hand, foot, and mouth disease in Ningbo, China, 2008–2011. J. Clin. Virol..

[6] Han J.Y., Jeong H.I., Park C.W., Yoon J., Ko J., Nam S.J., Lim B.K. (2018). Cholic Acid Attenuates ER Stress-Induced Cell Death in Coxsackievirus-B3 Infection. J. Microbiol. Biotechnol..

[7] Zhang Y.Y., Li J.Y., Xia H.H.X., Zhang S.L., Zhong J., Wu Y.Y., Miao S.K., Zhou L.M. (2013). Protective effects of losartan in mice with chronic viral myocarditis induced by coxsackievirus B3. Life Sci..

[8] Kim B.K., Kim J.H., Kim N.R., Lee W.G., Lee S.D., Yun S.H., Jeon E.S., Kim Y.C. (2012). Development of anti-coxsackievirus agents targeting 3C protease. Bioorg. Med. Chem. Lett..

[9] Fechner H., Pinkert S., Geisler A., Poller W., Kurreck J. (2011). Pharmacological and biological antiviral therapeutics for cardiac coxsackievirus infections. Molecules.

[10] Thibaut H.J., De Palma A.M., Neyts J. (2012). Combating enterovirus replication: State-of-the-art on antiviral research. Biochem. Pharmacol..

[11] Qiu F.Z., Shen X.X., Li G.X., Zhao L., Chen C., Duan S.X., Guo J.Y., Zhao M.C., Yan T.F., Qi J.J. (2018). Adenovirus associated with acute diarrhea: A case-control study. BMC Infect. Dis..

[12] Engelmann I., Coiteux V., Heim A., Magro L., Dewilde A., Dulery R., Hober D., Yakoub-Agha I. (2016). Severe adenovirus pneumonia followed by bacterial septicaemia: Relevance of co-infections in allogeneic hematopoietic stem cell transplantation. Infect. Disord. Drug Targets.

[13] Tan D.Y., Zhu H.D., Fu Y.Y., Tong F., Yao D.Q., Walline J., Xu J., Yu X.Z. (2016). Severe community-acquired pneumonia caused by human adenovirus in immunocompetent adults: A multicenter case series. PLoS ONE.

[14] Zhang S.Y., Luo Y.P., Huang D.D., Fan H., Lu Q.B., Wo Y., Chen G., Zhang X.A., Li Y., Tong Y.G. (2015). Fatal pneumonia cases caused by human adenovirus 55 in immunocompetent adults. Infect. Dis..

[15] Xie L.Y., Zhang B., Xiao N.G., Zhang F., Zhao X., Liu Q., Xie Z.P., Gao H.C., Duan Z.J., Zhong L.L. (2018). Epidemiology of human adenovirus infection in children hospitalized with lower respiratory tract infections in Hunan, China. J. Med. Virol..

[16] De Clercq E. (2011). The clinical potential of the acyclic(and cyclic)nucleoside phosphonates: The magic of the phosphonate bond. Biochem. Pharmacol..

[17] Hostetler K.Y. (2009). Alkoxyalkyl prodrugs of acyclic nucleoside phosphonates enhance oral antiviral activity and reduce toxicity: Current state of the art. Antivir. Res..

[18] Naesens L., Lenaerts L., Andrei G., Snoeck R., Van Beers D., Holy A., Balzarini J., De Clercq E. (2005). Antiadenovirus activities of several classes of nucleoside and nucleotide analogues. Antimicrob. Agents Chemother..

[19] Tang Z.Z., Zang N., Fu Y.X., Ye Z.X., Chen S.S., Mo S., Ren L., Liu E. (2018). HMGB1 mediates HADV7 infection-induced pulmonary inflammation in mice. Biochem. Biophys. Res. Commun..

[20] SÁ A.G.A., de Meneses A.C., de Araújo P.H.H., de Oliveira D. (2017). A review on enzymatic synthesis of aromatic esters used as flavor ingredients for food, cosmetics and pharmaceuticals industries. Trends Food Sci. Technol..

[21] Adams T.B., Cohen S.M., Doull J., Feron V.J. (2005). The FEMA GRAS assessment of benzyl derivatives used as flavor ingredients. Food Chem. Toxicol..

[22] US Food and Drug Administration (2011). Food additives permitted for direct addition to food for human consumption, flavoring agents and related substances. Code Fed. Regul..

[23] JECFA (2007). Safety Evaluation of Certain Food Additives and Contaminants: Prepared by the Seventy Seventh Meeting of the Joint FAO/WHO Ex-pert Committee on Food Additives (JECFA). http://apps.who.int/iris/bitstream/10665/43645/1/9789241660587_eng.pdf.

[24] Pavela R. (2015). Essential oils for the development of eco-friendly mosquito larvicides: A review. Ind. Crop. Prod..

[25] Zhang Q., Wang Y., Yang T.T., Li L., Li D. (2015). Palladium catalyzed ortho-C–H-benzoxylation of 2-arylpyridines using iodobenzene dibenzoates. Tetrahedron Lett..

[26] Kim B.-K., Cho J.-H., Jeong P., Lee Y., Lim J.J., Park K.R., Eom S.H., Kim Y.-C. (2015). Benserazide, the first allosteric inhibitor of Coxsackievirus B3 3C protease. FEBS Lett..

[27] Hoeben R.C., Uil T.G. (2013). Adenovirus DNA Replication. Cold Spring Harb Perspect. Biol..

[28] Marrugal-Lorenzo J.A., Serna-Gallego A., Berastegui-Cabrera J., Pachón J., Sánchez-Céspedes J. (2019). Repositioning Salicylanilide Anthelmintic Drugs to Treat Adenovirus Infections. Sci. Rep..

[29] Zhong D.W., Liu M.M., Cao Y., Zhu Y.L., Bian S.H., Zhou J.Y., Wu F.J., Ryu K.C., Zhou L., Ye D.Y. (2015). Discovery of Metal Ions Chelator Quercetin Derivatives with Potent Anti-HCV Activities. Molecules.

[30] Tsuchiya Y., Shimizu M., Hiyama Y., Itoh K., Hashimoto Y., Nakayama M., Horie T., Morita N. (1985). Antiviral activity of natural occurring flavonoids in vitro. Chem. Pharm. Bull..

[31] Chen T.C., Weng K.F., Chang S.C., Lin J.Y., Huang P.N., Shih S.R. (2008). Development of antiviral agents for enteroviruses. J. Antimicrob. Chemother..

[32] Bedard K.M., Semler B.L. (2004). Regulation of picornavirus gene expression. Microbes Infect..

[33] Farkas T., Yang K., Le Pendu J., Baines J.D., Cardin R.D. (2019). The Coxsackievirus and Adenovirus Receptor, a Required Host Factor for Recovirus Infection, Is a Putative Enteric Calicivirus Receptor. J. Virol..

[34] Kitazato K., Wang Y., Kobayashi N. (2007). Viral infectious disease and natural products with antiviral activity. Drug Discov. Ther..

[35] Grosso F., Stoilov P., Lingwood C., Brown M., Cochrane A. (2017). Suppression of Adenovirus Replication by Cardiotonic Steroids. J. Virol..

[36] Li Y.H., Tang S., Li Y.H., Cheng X.Y., Zhang X., Wang Y.X., Su F., Song D.Q. (2017). Novel 12N-substituted matrinanes as potential anti-coxsackievirus agents. Bioorg. Med. Chem. Lett..

[37] Zhai X., Qin Y., Chen Y., Lin L., Wang T., Zhong X., Wu X., Chen S., Li J., Wang Y. (2016). Coxsackievirus B3 induces the formation of autophagosomes in cardiac fibroblasts both in vitro and in vivo. Exp. Cell Res..

[38] Feld J.J., Jacobson I.M., Sulkowski M.S., Poordad F., Tatsch F., Pawlotsky J.M. (2017). Ribavirin Revisited in the Era of Direct-Acting Antiviral Therapy for Hepatitis C Virus Infection. Liver Int..

[39] Markland W., McQuaid T.J., Jain J., Kwong A.D. (2000). Broadspectrum antiviral activity of the IMP dehydrogenase inhibitor VX-497—a comparison with ribavirin and demonstration of antiviral additivity with alpha interferon. Antimicrob. Agents Chemother..

[40] Ning Q., Brown D., Parodo J., Cattral M., Gorczynski R., Cole E., Fung L., Ding J.W., Rotstein O., Phillips M.J. (1998). Ribavirin inhibits viral-induced macrophage production of TNF, IL-1, the procoagulate fg12 prothrombinase and preserves the Thi cytokines production but inhibits Th2 cytokines response. J. Immunol..

[41] Crotty S., Cameron C.E., Andion R. (2001). RNA virus error catastrophe: Direct molecular test by using ribavirin. PNAS.

[42] Reed L.J., Muench H. (1938). A simple method of estimating fifty percent endpoints. Am. J. Hyg..

[43] Kumar P., Nagarajan A., Uchil P.D. (2018). Analysis of Cell Viability by the MTT Assay. Cold Spring Harb Protoc..

